# Effectiveness of an online group course for adolescents and young adults with depressive symptoms: study protocol for a randomized controlled trial

**DOI:** 10.1186/1745-6215-12-196

**Published:** 2011-08-19

**Authors:** Rianne AP van der Zanden, Jeannet JAM Kramer, Pim Cuijpers

**Affiliations:** 1 Trimbos Institute, Netherlands Institute of Mental Health and Addiction, Da Costakade 45, Postbox 725, 3500 AS, Utrecht, The Netherlands; 2 Department of Clinical Psychology, EMGO Institute for Health and Care Research, VU University, Van der Boechorststraat 1, 1081 BT Amsterdam, The Netherlands

## Abstract

**Background:**

Depression is a common condition whose first onset is usually in late adolescence or early adulthood. Internet-based interventions are an effective treatment approach to depression. The aim of this study is to investigate the effectiveness of a Dutch online cognitive-behavioural group course known as Master Your Mood (*Grip op Je Dip*) for young people reporting depressive symptoms. Secondary research questions involve maintenance of effect at 6 months, mediators, and predictors of better outcomes.

**Methods:**

We will conduct a randomised controlled trial (RCT) in which 244 young people aged 16-25 are randomly allocated to the Grip op Je Dip (GOJD) online group course or to a waiting list control group. The participants will be recruited from the general population. The primary outcome measure will be the severity of depressive symptoms according to the Center for Epidemiological Studies Depression Scale (CES-D). Other outcomes will include anxiety (Hospital Anxiety and Depression Scale-Anxiety, HADS) and mastery (Mastery Scale). Assessments will take place in both groups at baseline and three months later. Effect maintenance will be studied in the GOJD group six months after baseline, with missing data imputed using the expectation-maximisation method. Mediators and predictors of better outcomes will also be identified.

**Discussion:**

The trial should add to the body of knowledge on the effectiveness of Internet-based interventions for depression. To our knowledge, this will be the first RCT on an online group intervention in this field.

**Trial registration:**

NTR1694

## Background

Depression is a common mental disorder among adolescents and young adults. One recent study found a 12-months prevalence of 6.7% of 18- to 25-year-olds [1]. First onset is usually in adolescence; by 18 years of age, up to one in every four adolescents has had at least one depressive episode ([2-4]. Depression early in life can have serious implications for individuals' school and professional careers [5]. Adolescent depression is associated with problems such as poor school performance, school absence and dropout [6], problematic relations with parents and peers [7], increased use of tobacco and alcohol [8] and suicidal behaviour [9].

Subclinical depression is also a common condition, with an estimated prevalence of 17% to 21% among Dutch adolescents [10,11]. It involves having some depressive symptoms which together do not meet the full DSM-IV criteria for major depression [12]. Subclinical depression has been found to be a strong predictor of the onset of major depressive disorder within the next year [13,14]. There are also indications that the psychosocial functional impairment of people with subclinical depression is comparable to that experienced by people diagnosed with major depression [15,16]. Beyond the personal suffering involved, both major and subthreshold depression impose significant economic burdens in terms of health care costs and production losses in paid and unpaid work [17,18].

In view of the high prevalence rates, the serious outcomes and the economic costs of depression, it is essential to intervene at an early stage. Yet young people are not inclined to seek professional help. They tend to deny or underestimate problems, question the benefits of help and fear stigmatisation [19]. And if they do seek help, they often encounter waiting lists [20].

Internet-based approaches may offer a solution for the stigmatisation problem, in that they provide anonymity and the opportunity to undergo the intervention in the privacy of home. Another benefit is to enable a reduction in contact hours between professionals and clients. This could help tackle the problem of waiting lists and shortages of therapists. Internet-based interventions, with or without professional support and mostly based on cognitive-behavioural principles, have been found effective in treating depression, with results comparable to traditional psychological approaches [21-24].

The current study will focus on one specific type of Internet-based intervention: a professionally facilitated, cognitive-behavioural group course designed for young people with symptoms of depression. The perceived advantages of online group sessions as compared to individual approaches are social support and mutual recognition by group members (though they remain anonymous to one another) and the reduction of professional contact hours per participant as compared to individual treatment with support [25]. Three earlier studies on online group courses in mental health care have found positive outcomes in pre-post measurements, although relatively high attrition rates were reported, a general problem in Internet interventions [26].

Despite the clear benefits of web-based interventions for depression and their availability for all age groups from about 13 onwards, there is a lack of outcome studies on adolescents [27,28]. Only two randomised controlled trials have been conducted on prevention programmes for depression and anxiety [29,30]. The Calear study found that a universal prevention programme was effective in reducing symptoms of anxiety and depression only in males. The Van Voorhees study showed that a web-based behavioural change programme in primary care, in combination with either motivational interviewing or brief advice, was associated with declines in depressed mood and in the likelihood of clinical depression symptom levels; in combination with motivational interviewing it reduced the likelihood of depressive episodes and hopelessness.

The primary aim of the study we describe here will be to evaluate the effectiveness of a web-based group course called Grip op Je Dip (Master Your Mood), designed for young people aged 16 to 25 with depressive symptoms, by comparing it to a waitlisted control group. Secondary aims concern effect maintenance and potential predictors of positive outcomes. The present article describes and discusses the study design.

## Methods

### Study design

We will conduct a randomised controlled trial with two parallel groups: the GOJD online course group versus a waiting list control group. Ethical approval has been granted by an independent medical ethics committee (CCMO no. NL18984.097.07).

### Participating mental health care agencies

A total of 10 mental health care agencies have agreed to participate in the project. In providing the online GOJD course, all agencies work at a nationwide level with participants from all over the Netherlands.

### Sample

The sample is to consist of 242 young people aged 16-25 with mild to moderate depressive symptoms. The inclusion criteria are as follow: age 16 to 25, informed consent (including parental consent if aged 16 or 17) and a CES-D depression score from 10 to 45. The CES-D cut-off score for clinical depression is 16 [31] for adults, and for adolescents it is either 22 [32] or 24 [33]. For subclinical depression, no CES-D cut-off scores have been determined, but we have set the lower limit at 10 for this study. Applicants will be excluded if there are indications of suicidal ideation with intent and plan as assessed with the MINI-International Neuropsychiatric Interview (MINI-Plus) [34,35]. MINI-Plus assessment is mandatory for individuals with CES-D scores higher than 24; the MINI-Plus will be administered online in the chatroom.

### Recruitment

Participants will be recruited in the general population through advertisements in local and national newspapers, banners on websites, and leaflets provided in doctors' offices, mental health agencies and schools. Interested young people may apply for participation by completing a screening questionnaire on the website http://www.gripopjedip.nl. Those with CES-D scores between 10 and 45 will receive additional information about the study, an informed consent form and a baseline questionnaire. For those aged 16 or 17, parental consent is also required. Those with scores between 25 and 45 will be invited for an online session to assess suicidal ideation and plan using the MINI-Plus interview. Once judged eligible for the GOJD course, applicants will be randomly allocated to the course group or the waitlisted group (3 months).

### Randomisation

Randomisation will take place after completion of the screening procedure, informed consent statement and baseline questionnaire before the start of the course. Random allocation will be generated automatically by an online computer program so it cannot be influenced by course facilitators or researchers. A blocked randomisation scheme will be used (blocks of two), stratified by depressive symptoms (scores 10-24 versus 25-45) and age (younger than 18 versus 18 or older). These allocation sequences will be unknown to the course facilitators who are pushing the randomization button. Participants will be informed of their allocation by email and will receive a tailored referral if declined. Course participants will also receive a log-in code and homework for the first session.

### Conditions: the intervention

The GOJD online course derives from the face-to-face intervention of the same name developed by the Trimbos Institute (Netherlands Institute for Mental Health and Addiction). That intervention was based on the Dutch version of the Coping with Depression Course [36]. The face-to-face course was adapted to the Internet in a collaborative effort involving the Trimbos Institute and three mental health institutions [37].

The resulting online GOJD group course is a structured, psychoeducational form of cognitive-behavioural therapy for depression. The core focus is the cognitive restructuring of thinking patterns. Course participants are encouraged to detect their own unproductive, unrealistic thoughts, and they are then taught to transform these into realistic, helpful thoughts. Performance of pleasant daily activities is also encouraged, and a mood measure is filled in daily to help understand the connection between pleasant activities and mood level. The course is given in a secured chatroom that is part of the website http://www.gripopjedip.nl, which also provides information and video films about depression. Participants' anonymity is guaranteed by a self-chosen nickname. Only a mobile phone number must be given to facilitators for sending pre-session SMS reminders. The course makes use of text and figures, smileys to express feelings in written texts, and home exercises.

GOJD consists of six 90-minute sessions, conducted at a fixed time once a week. The course is facilitated by one or two trained mental health promotion workers, depending on group size (a maximum of 6) and their experience in conducting online courses. Facilitators receive two days' prior training in the technical and therapeutic conduct of the course and in administering the online MINI-Plus interview.

### Conditions: the waiting list

The wait listed group does not get an intervention. They are told by e-mail that they will receive an invitation to participate after the waiting period of 3 months.

### Support

After the screening procedure, the participants are in contact with the facilitator and the group members only during the weekly course sessions in the chatroom. They are allowed to seek additional help outside the course if they wish.

### Assessments

Assessments will take place before randomisation (t0), three months later (t1) and three months after t1 (t2). Participants in both groups will receive automated emails with invitations to complete online questionnaires. Subsequent email reminders will be sent five and ten days after the first invitation, if necessary. To encourage response, participants will receive 10 euros' compensation for completing the t1 questionnaire and 10 euros for the t2 questionnaire. If they complete both questionnaires, a 5-euro bonus will be added, totalling 25 euros.

### Instruments

#### Primary outcome

##### Depressive symptoms

Symptoms of depression will be assessed with the Center for Epidemiological Studies Depression Scale (CES-D) [31,38]. It measures the frequency of 20 depressive symptoms over the past week using 4-point Likert scales. The total score range is 0 to 60, with a higher rating meaning more depressive symptoms. Computerised and paper-and-pencil versions of the CES-D correlate at a very high level [39]. The web-based CES-D has proved a reliable and valid screening instrument in a Dutch adolescent population [32].

#### Secondary outcomes

##### Anxiety symptoms

The anxiety subscale of the Hospital Anxiety and Depression Scale (HADS) [40] will be used to assess anxiety symptoms. The Dutch version of the HADS has been validated [41]. The anxiety subscale consists of seven items measuring anxiety symptoms on a 4-point Likert scale, with a score range of 0 to 21, and a higher rating indicating a higher state of anxiety.

#### Perceived control

The Dutch version of the 5-item Mastery Scale will be used to assess perceived control [42]. The concept of mastery refers to beliefs about one's own ability to control one's environment. Responses are rated on a 5-point Likert scale, with a total score range of 5 to 25; a higher score indicates a greater sense of mastery. The Mastery Scale has good psychometric properties [42].

#### Additional measures

##### Motivation

At baseline we will assess motivation to take part in preventive group interventions, using four subscales of the Nijmegen Motivation List for Prevention (NML-P) [43]. The subscales are Readiness, Doubt, Support and Burden. Their reliability is acceptable, with Cronbach's alphas ranging from .80 to .84.

#### Other information

At baseline we will also assess demographic information and any previous or present receipt of professional help for psychological problems. Follow-up assessments will record the use of professional help (t1 and t2) and participants' evaluations of the intervention (t1, in course group only).

Course adherence will be measured in terms of session attendance and completion of homework assignments. Session attendance will automatically be recorded when the participant enters the chatroom. Homework completion will be recorded by the facilitators in an online logbook.

### Sample size

The trial is powered to detect a clinical effect size of *d *= 0.32 or larger in a one-sided test (alpha = .05) at a power of 80% (1-β). The hypothesis is directional, with better outcomes expected for the course group. A total of 242 participants are needed in the study, n = 121 per condition.

### Statistical analyses

To assess whether the randomisation results in two comparable groups at baseline, and whether differential loss to follow-up later occurs, we will use t-tests, chi-square tests and logistic regression (p < .10). Analyses with regard to effectiveness will be based on the intention-to-treat principle.

Missing data at t1 and t2 will be imputed using the expectation-maximisation (EM) method, as implemented in SPSS Missing Value Analysis. It imputes missing values by maximum likelihood estimation using the observed data in an iterative process [44]. All randomised participants will be included in the analyses in line with their allocation, regardless of how many sessions they complete (intention-to-treat principle).

Since the online GOJD is a group course, some subjects will be participating in the same course group, leading to some amount of clustering. The clustering would violate the assumption of independence of observations and might thus affect standard errors and *P*-values. We will therefore use linear regression models, controlling for data clustering, in analysing post-test (t1) between-group differences on the continuous outcome measures. Robust standard errors and correct *P-*values will be obtained using the first-order Taylor-series linearisation method, as implemented in Stata.

Magnitudes of intervention effects will be estimated using Cohen's *d *[45]. Effect sizes will first be calculated for each condition separately by subtracting the mean post-test score from the mean pre-test score and dividing the result by the standard deviation at pre-test. The effect size of the comparison group will then be subtracted from that of the experimental group. A difference in *d *0.5 would indicate that the experimental group mean is half a standard deviation greater than the control group mean. For Cohen's *d*, an effect size of 0.2 to 0.3 may be regarded as a small effect, around 0.5 as a medium effect and 0.8 to infinity as a large effect.

To determine effect maintenance at 6 months in the experimental group, we will use the follow-up (t2) assessment data. The number of participants showing reliable, clinically significant change on the CES-D from pre-intervention to post-intervention and follow-up will be calculated using the method of Jacobson and Truax [46]. To find predictors for more or less successful intervention outcomes, we will use regression analyses to study effect modification. The individual standardised change scores (effectsizes; pre- to post-intervention) will serve as the outcome measure, and interaction terms between the treatment dummy and participants' characteristics will be included as predictors, along with their constituent main effects. Predictors for successful and unsuccessful outcomes will be demographic variables such as age, gender and educational attainment and course-related variables such as web-chatting experience and motivation to take part in preventive group interventions [43].

To detect possible dose-response relationships in respondents randomised to the course, we will use a linear model to regress the primary outcome (depressive symptoms) on the number of sessions they attended. Tests will be conducted at alpha = .05 with 95% confidence intervals.

## Discussion

The study described here is a randomised controlled trial in which young people aged 16 to 25 with depressive symptoms will take the GOJD (Master Your Mood) online group course; the outcomes will be compared to those of a waiting list control group. The primary aim is to assess the effectiveness of the online course in terms of severity of depressive symptoms. Secondary aims are to study effect maintenance and to identify predictors of positive outcomes.

Internet-based interventions are known to be effective in addressing depression. By virtue of their anonymity and the reduced need for contact between participants and professionals, they hold promise to reach many individuals who need them. In the field of web-based interventions for depression, studies with young people are still scarce, as are studies on online group courses. This trial should add to the body of knowledge on the effectiveness of such interventions. We shall now discuss some methodological issues in the current trial.

A strong feature of the current trial is that the recruitment and screening of participants and the inclusion and exclusion criteria are similar to the usual procedures in mental health agencies. This should enhance the external validity of the results. The main differences between the study and routine care are the randomisation and the informed consent required for the study. In addition, in routine care, participants with CES-D scores higher than 45 may be allowed to participate in the course, if only in exceptional cases in pressing circumstances and under specific conditions.

One limitation of the study concerns the impossibility of comparing the GOJD outcomes with those of the waitlisted group at the six-month follow-up, since the latter will receive access to the course three months after baseline. This means that the longer-term effectiveness of the course cannot be assessed in this study. We will, however, study effect preservation at six months in the GOJD group. Another study limitation is that the blinding of participants to experimental conditions is not feasible, because of the behavioural nature of the intervention.

A point of discussion is how far results can be generalised. To minimise selection bias, we will recruit participants via a broad range of channels: advertising in local and national newspapers, website banners, and leaflets in doctors' offices, mental health agencies and schools. Each participant's recruitment channel will be recorded; the distribution of the various channels will determine the degree of generalisability. Selection bias could also arise from personality traits of people attracted to a group intervention; these might differ from the traits of those who prefer individual therapy or no help at all.

A final limitation is that study participants will not be diagnosed. They will be selected on the basis of self-rated instruments, together with a diagnostic interview (MINI-Plus) in the chatroom, which is used as a screener to exclude applicants with suicidal tendencies. This means that the current study will be difficult to compare with studies in which participants have been diagnosed. The results will therefore not be generalisable to people with a DSM diagnosis of depression.

## Competing interests

Rianne van der Zanden is one of the developers of the online GOJD group course and the Trimbos Institute is co-owner of the GOJD intervention, but we derive no financial income from the GOJD website or the group course.

## Authors' contributions

RZ conceived the project, did the acquisition of funding, set up the study design and performed the main contribution to the draft. JK participated in the acquisition of funding and in the design of the study and had a substantial contribution to the draft. PC participated in the design of the study and revised the draft critically. All authors read and approved the final manuscript.
